# Standardisation of natural dye powders *via* spray drying: initial physicochemical characterisation

**DOI:** 10.1039/d6ra04201e

**Published:** 2026-08-28

**Authors:** Adhi Kusumastuti, Samsudin Anis, Octavianti Paramita, Retno Marlangen, Ooi Boon Seng

**Affiliations:** a Faculty of Engineering Universitas Negeri Semarang, Kampus UNNES Sekaran Gunungpati 50229 Semarang Indonesia adhi_kusumastuti@mail.unnes.ac.id; b School of Chemical Engineering, Engineering Campus Universiti Sains Malaysia 14300 Nibong Tebal Pulau Pinang Malaysia

## Abstract

Liquid natural dyes exhibit some drawbacks in terms of storage, distribution, dosage control, and quality consistency. This study aimed to evaluate the initial standardisation of natural dye powders from the bark of Tingi (*Ceriops tagal*), wood of Tegeran (*Cudrania javanensis*), and leaves of *Indigofera* (*Indigofera tinctoria*) through a spray drying process with the addition of 15% (w/w) maltodextrin as a physical matrix. The resulting powders were characterised in terms of yield, colour strength (UV-vis spectroscopy), molecular composition (FTIR spectroscopy), solid-state structure (XRD), moisture content, water activity, particle size distribution, and morphology (SEM). The study achieved a yield of 72.33–85.67%, with the highest yield obtained by Tingi. The UV-vis comparison of fresh extract and reconstituted powder indicated that despite the intensity changes and peak shifts experienced by some samples, the main absorption characteristics after spray drying were still observable. The FTIR spectrum showed the existence of main functional groups related to the dye components and maltodextrin, while the XRD patterns showed dominant amorphous or semi-amorphous characteristics. These findings indicate the possibility of a physical association between the pigment and the maltodextrin matrix, albeit not used as definitive evidence of hydrogen bond formation. The moisture contents were in the range of 9.87–11.27%, with water activity of 0.60–0.64, demonstrating the initial moisture-related risks. The PSD and SEM analyses implied the differences in the size characteristics and particle morphology among the dyes. Overall, this study provides initial information regarding the physicochemical characteristics of maltodextrin-aided spray-dried powder. Further evaluation of storage stability and dyeing performance is necessary.

## Abstract 2

Liquid natural dyes have several limitations related to storage, distribution, dosage control, and quality consistency.

## Graphical abstract

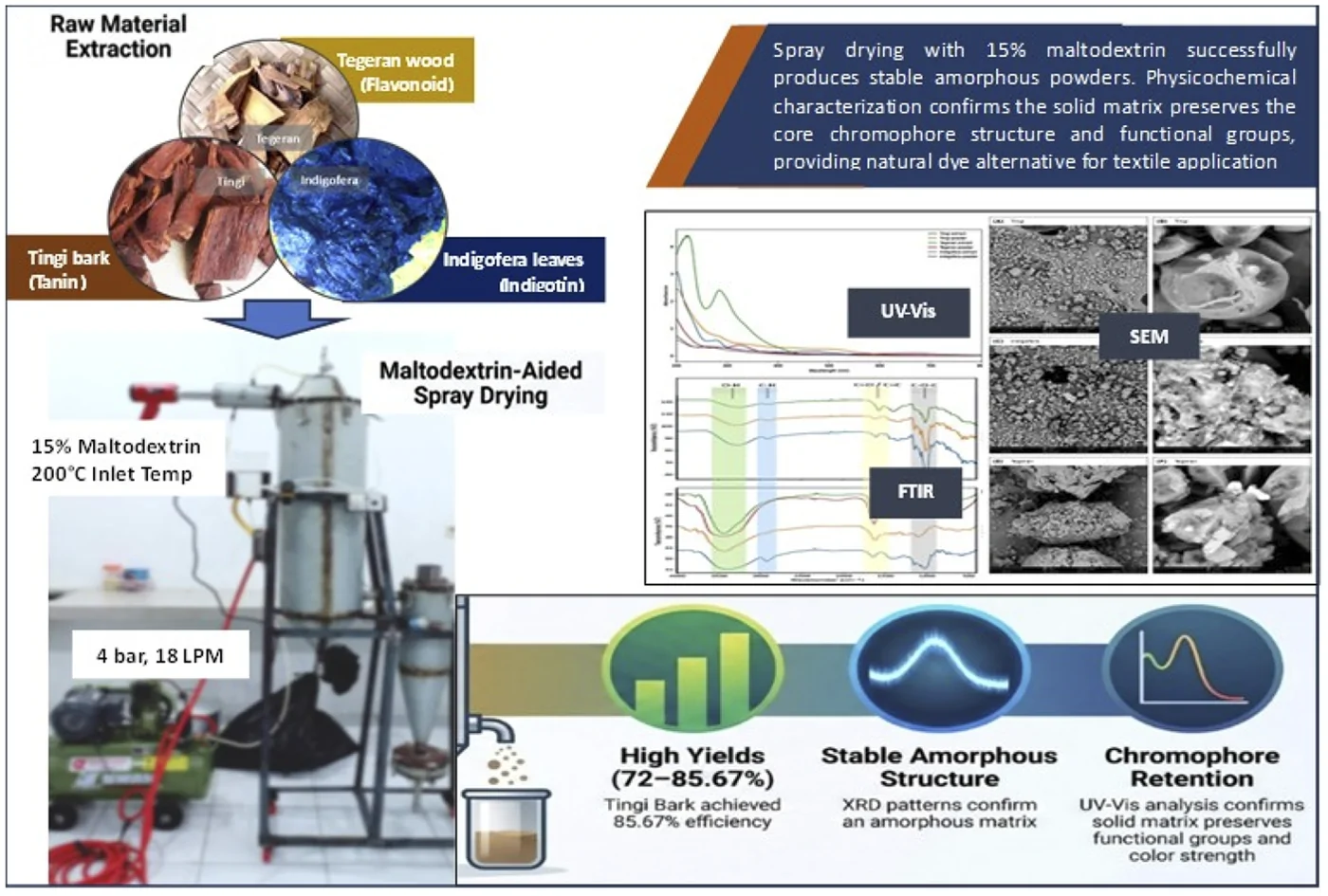

## 1 Introduction

Dyes are widely used in various industries, such as textiles, food, pharmaceuticals, cosmetics, crafts, and leather tanning. In general, these industries utilize synthetic dyes because they offer several advantages, such as easy market availability, guaranteed colour availability, a wide variety of colours, greater practicality or ease of use, and greater economic value.^1^ However, synthetic dyes are carcinogenic.^2–4^ This condition encourages technological innovation and accelerates the transition to more environmentally friendly textile manufacturing processes.

Growing consumer awareness of environmentally friendly products and the need to preserve the environment have led to a global increase in the use of natural dyes over the past two decades.^5,6^ This enables the production of environmentally friendly textile materials.^7,8^ The application of natural dyes is highly feasible in Indonesia due to the abundance of natural dye sources. However, the use of natural dyes is facing major obstacles in the form of a relatively long extraction process, low colour reproducibility, and inefficient processing costs.^9,10^

Various studies have been conducted to obtain high-quality natural dyes through optimization of extraction methods,^11,12^ fabric surface modification,^13,14^ and dyeing methods.^2,15^ In general, although extraction efficiency and colour fastness can be achieved at acceptable levels, the problem of colour consistency between batches remains a major challenge, especially since natural dyes are generally used in the form of unstable liquid solutions. In addition, the liquid form complicates distribution, storage, and dose control on an industrial scale. Therefore, the development of standardization techniques for natural dyes is an urgent need to improve the practicality and reliability of their application. Converting liquid extracts of natural dyes into powders can be a solution to the impracticality of using natural dyes.^16–18^

The literature shows that various crystallization techniques have been studied, including tank crystallizers,^19^ surface crystallizers, circulating magma vacuum crystallizers, forced circulation liquid evaporator crystallizers, continuous laminar shear crystallizers,^20^ and continuous stirred tank reactor crystallizers.^21^ In general, crystallization processes can be divided based on their operation methods, including phase transformation approaches such as spray drying for powder formation,^22^ vacuum drying,^23^ and freeze drying.^24^ Previous research has developed a natural dye powder crystallization device based on spray dryer technology, which converts liquids into powder form.^25,26^ This device uses a semi-crossflow drying system, which uses liquid droplets and hot air. The main challenge in the development of natural dyes is not only converting the liquid extract into powder but also obtaining measurable and comparable initial physicochemical characteristics. The standardisation involves process efficiency, moisture content, chromophore stability, functional group integrity, and particle size distribution to ensure suitability for textile application. In this context, spray drying with maltodextrin as a physical carrier was used as an approach to generate stable, manageable and reliable natural dye powders.^27,28^ The study aimed to develop and evaluate the initial standardisation of natural dye powder produced by spray drying with 15% maltodextrin as a physical matrix for further development in textile dyeing applications. However, its compatibility in textile dyeing needs to be confirmed through textile performance tests. Maltodextrin was incorporated prior to drying to prevent clumping, improve powder flowability, and protect thermally sensitive compounds during the drying process, without acting as an active colouring agent.^29^ Although the maltodextrin-aided spray drying process is widely used for natural dyes, most studies have focused on one type of material or the optimisation of carrier composition.^30–33^ This study is unique in comparatively evaluating three local natural dye sources with different pigment characteristics. Tannin-based dyes of *Tingi*, flavonoid-based dyes of *Tegeran*, and indigotin-based dyes of *Indigofera* were prepared under the same conditions. This study emphasises the initial standardisation of natural dye powders by yield analysis, colour strength analysis, molecular composition, water content, water activity, particle size distribution, and morphology.

Although natural dyes are generally safer than synthetic dyes, a consideration of the safety aspect is inevitable. The natural dyes used in this study, *i.e. Tingi* bark (*Ceriops tagal*), *Tegeran* wood (*Cudrania javanensis*), and *Indigofera* leaves (*Indigofera tinctoria*), are derived from natural ingredients that have been extensively used in traditional textile dyeing.

However, the chromophores present in natural dyes, such as polyphenols and tannins, can exhibit certain biological activities that should be considered in relation to product quality. Therefore, this research was carried out through several steps, including extraction, filtration and encapsulation. These processes are expected to reduce impurities and improve the stability of the end products.

Moreover, some previous studies have revealed that the utilisation of natural dyes in textile application is relatively safe with lower health risks than that of synthetic dyes. Therefore, the utilisation of natural dyes in this research could be considered as an environmentally friendly alternative, although further toxicological studies are required for broader application.

## 2 Method

This study was designed to evaluate the stability and quality of natural dye powder produced by spray drying with the addition of 15% (w/w) maltodextrin as a physical matrix. The maltodextrin concentration of 15% (w/w) was determined based on a previous study on the formulation of natural dye powder and maltodextrin-aided pigment systems.^34^ This research method was planned systematically to ensure that the results obtained represent real conditions relevant to textile industry applications. An experimental approach was chosen because it allowed for strict control of process variables, and the relationship between encapsulant concentration, pigment characteristics, and product quality parameters could be identified clearly and measurably.

The research procedure involved three main stages: (1) preparation of the dye extract solutions, (2) spray drying to produce dye powder, and (3) analysis of physicochemical properties, including UV-vis absorption spectra as an indicator of colour retention. Moisture content and water activity were used to describe the initial moisture-related characteristics of the spray-dried powders, rather than as direct indicators of long-term storage stability. This approach not only focused on the characteristics of the final product but also provided a mechanistic understanding of pigment protection by the maltodextrin matrix during the drying process.

This method was chosen because spray drying, a commonly used technique in the food, pharmaceutical, and textile dye industries, is known for its efficiency and ability to produce powders. This method could convert natural dye extract into powder that is more practical for handling, dose measurement, and distribution. In this study, moisture content and water activity were used as initial parameters to describe the characteristics of residual water in the powder. Both parameters showed the potential risks of humidity such as caking, stickiness, and decreased flowability.^35^ UV-vis analysis was chosen because it provides quantitative information on the stability of pigment chromophores and allows direct comparison with standard samples.^36,37^

This research applied a completely randomized single-factorial design to evaluate the effect of natural dye type on the physicochemical characteristics of the obtained powder. The independent variable of this study was the types of natural dyes, *i.e. Tingi* bark (*Ceriops tagal*), *Tegeran* wood (*Cudrania javanensis*), and *Indigofera* leaves (*Indigofera tinctoria*).

All samples were proceeded under controlled conditions. The extraction method was considered based on the materials, in which *Tingi* and *Tegeran* dyes were extracted through the boiling method, while *Indigofera* was extracted through the fermentation method to ensure optimal pigment. After the extraction process, all samples were proceeded under the same conditions, involving maltodextrin addition (15%) and drying process parameter. Thus, the resulting powder was determined by the intrinsic characteristics of each dye. To assure data reliability, each treatment was performed 3 times. The parameters analysed were yield, absorbance, particle size distribution, water content, and FTIR absorption characteristics at the representative absorption band. Statistical analysis was also applied as a comparative instrument between the groups of dyes through ANOVA and the Kruskal–Wallis test for parametric and non-parametric analysis, respectively, at a significance level of 0.05.

### 2.1. Materials

The main materials used in this study included natural dye extracts: *Tingi* bark (*Ceriops tagal*), *Tegeran* wood (*Cudrania javanensis*), and *Indigofera* (*Indigofera tinctoria*). Maltodextrin (DE 8–15) was obtained from Sigma-Aldrich and used as an encapsulating agent. Maltodextrin with this DE range was selected due to its suitable properties, including moderate hygroscopicity, good solubility, and film-forming ability, which are beneficial for spray drying applications. Other chemicals included distilled water, ethanol (technical grade, 96%), and phosphate buffer solution (pH 6.8) to maintain pH stability throughout the process.

### 2.2. Solution preparation and spray drying process

The feed solution was prepared by dispersing 200 g of *Indigofera* paste into 740 g of distilled water at room temperature. Dispersion was carried out using a mechanical stirrer at a medium speed for 15 minutes to obtain a homogeneous and agglomeration-free suspension. The *Indigofera* paste used was in the form of oxidized indigo, and this process aimed to obtain an even particle distribution. Next, 90 g of maltodextrin (15% w/w) was gradually added to the suspension as a carrier, with continuous stirring until the maltodextrin was hydrated and completely dispersed. After all the components were homogeneously mixed, the solution volume was adjusted to reach a total mass of approximately 1000 g, and the solution was allowed to stand until the viscosity was stabilized. The indigo feed suspension was pre-filtered using a stainless steel mesh (100 µm) to remove coarse impurities and agglomerates prior to spray drying.

The extraction of natural dyes from the *Tingi* bark and *Tegeran* wood was carried out by cleaning each raw material to remove dirt and then crushing it to increase the surface area in contact with the solvent. Each material was weighed and mixed with water as a solvent with a raw material to solvent ratio of 1 : 8 (w/v). The mixture was extracted by boiling until it reached the boiling temperature and was maintained at that temperature while stirring periodically to ensure a homogeneous extraction process. The boiling process continued until the solution volume was reduced to approximately half of its initial volume. After boiling was complete, each extract solution was cooled to room temperature and filtered to separate the solid residue from the solution. The filtrate obtained was the *Tingi* dye extract and the *Tegeran* dye extract with around 1.2% (w/v) soluble solid content, which were then used in the spray drying process. Each extract was then mixed with maltodextrin (15% w/w) with continuous stirring to ensure that maltodextrin was completely dispersed. The selection of maltodextrin at 15% (w/w) was based on considerations of the balance between pigment protection, physical stability of the powder, and readiness for application in textile processes. The addition of maltodextrin into the solution provided feed solution viscosities of around 4.5 cSt, 3.7 cSt, and 5.2 cSt for *Tingi* bark, *Tegeran* wood, and *Indigofera*, respectively.

The powdering process of the dye solution was carried out using a spray dryer. The extracted dye solution was first homogenized and then flowed into the spray dryer system using a feed pump with a flow rate of 800 mL per hour. The solution was atomized through an air-pressurized nozzle (0.6 mm size) with a fogging pressure of 4 bar and a fogging air flow rate of 18 L per minute to form fine droplets. Hot air was introduced into the drying chamber at an inlet temperature of 200 °C and a flow velocity of 4 m s^−1^ to provide the heat energy required for the drying process. During the process, direct contact between the solution droplets and the hot air caused rapid evaporation of the solvent, resulting in the formation of dye particles in the form of a dry powder. The resulting powder was then separated from the air stream using a cyclone system and collected as the final product, while the exhaust air at a temperature of around 55–57 °C was released through the exhaust duct. The resulting dye powder was then stored in airtight glass bottles with silica gel at room temperature for further characterization and application.

### 2.3. UV-vis spectrum analysis

Absorption spectra were measured using a UV-vis spectrophotometer (Shimadzu UV-2600) at a wavelength of 200–800 nm with a quartz cuvette (1 cm path length). The powder samples were dissolved in distilled water at a concentration of 1 g/100 mL and stirred until homogeneous before analysis. The spectra of fresh dye extracts before spray drying were used as the reference spectra and compared with the reconstituted spray-dried powder spectra. The measurement was performed following the standard spectrophotometric procedures commonly used for dye characterization.

UV-vis analysis was carried out to compare the spectral characteristics of the fresh dyes before spray drying and the reconstituted spray-dried powder. The measurement was performed in the wavelength range of 200–800 nm. The comparison was based on the *λ* maximum, maximum absorbance, and ratio of absorbance retention at the characteristic wavelength.^38^ The absorbance retention was determined using the following equation:
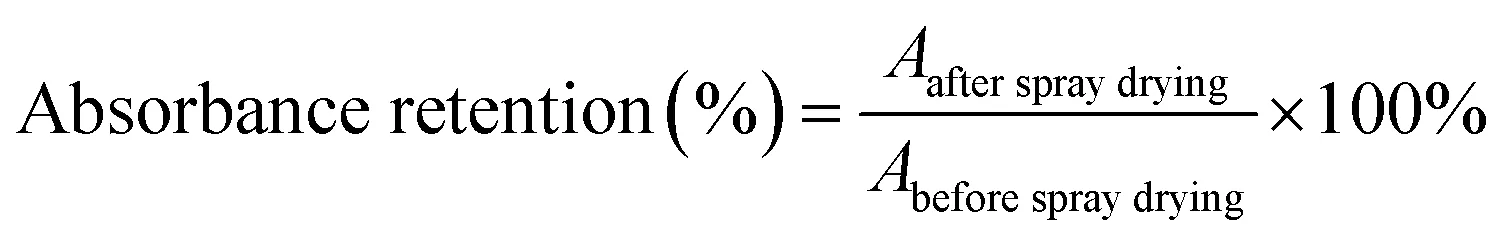


The retention value was used as an indicator of the changes in spectral characteristics after the spray drying process rather than as a single evidence of chromophore chemical stability.

### 2.4. Molecular composition determination

PerkinElmer Spectrum IR software (version 10.6.2) was used to determine the molecular composition of the natural dye powder sample by quantifying the absorption of infrared radiation at various wavelengths. It enables the identification of functional groups and provides information about the molecular structure by determining the presence and intensity of certain peaks. FTIR analysis was conducted following standard procedures for functional group identification in organic and polymeric materials.

### 2.5. Moisture content determination

The moisture content, which represents the free water content remaining after the spray drying process, was analysed using a Shimadzu Moisture Analyzer (MOC63u). Approximately 5 g of the sample was weighed into an analytical dish and heated at 120 °C until a constant weight was reached. Results are reported as a percentage (%) of wet weight. This method is based on the gravimetric principle of moisture determination, which is widely applied in accordance with standard methods such as AOAC.

### 2.6. Particle size analysis

The measurement of the particle size distribution of spray-dried powder was carried out using a laser diffraction-based Microtrac Particle Size Analyzer with a wet dispersion method. The dye powder sample was first dispersed in a solvent until a homogeneous dispersion was achieved and then inserted into the instrument's measuring cell. During the measurement, the dispersed particles were passed through a laser beam and the light scattering pattern was analysed to obtain a volume-based particle size distribution. The measurement results were reported in the form of a particle size distribution curve and characteristic parameters such as D10, D50, and D90. The analysis was conducted using laser diffraction principles, which are widely accepted as a standard method for particle size characterization.

## 3 Results and discussion

### 3.1. Yield

**Fig. 1:**
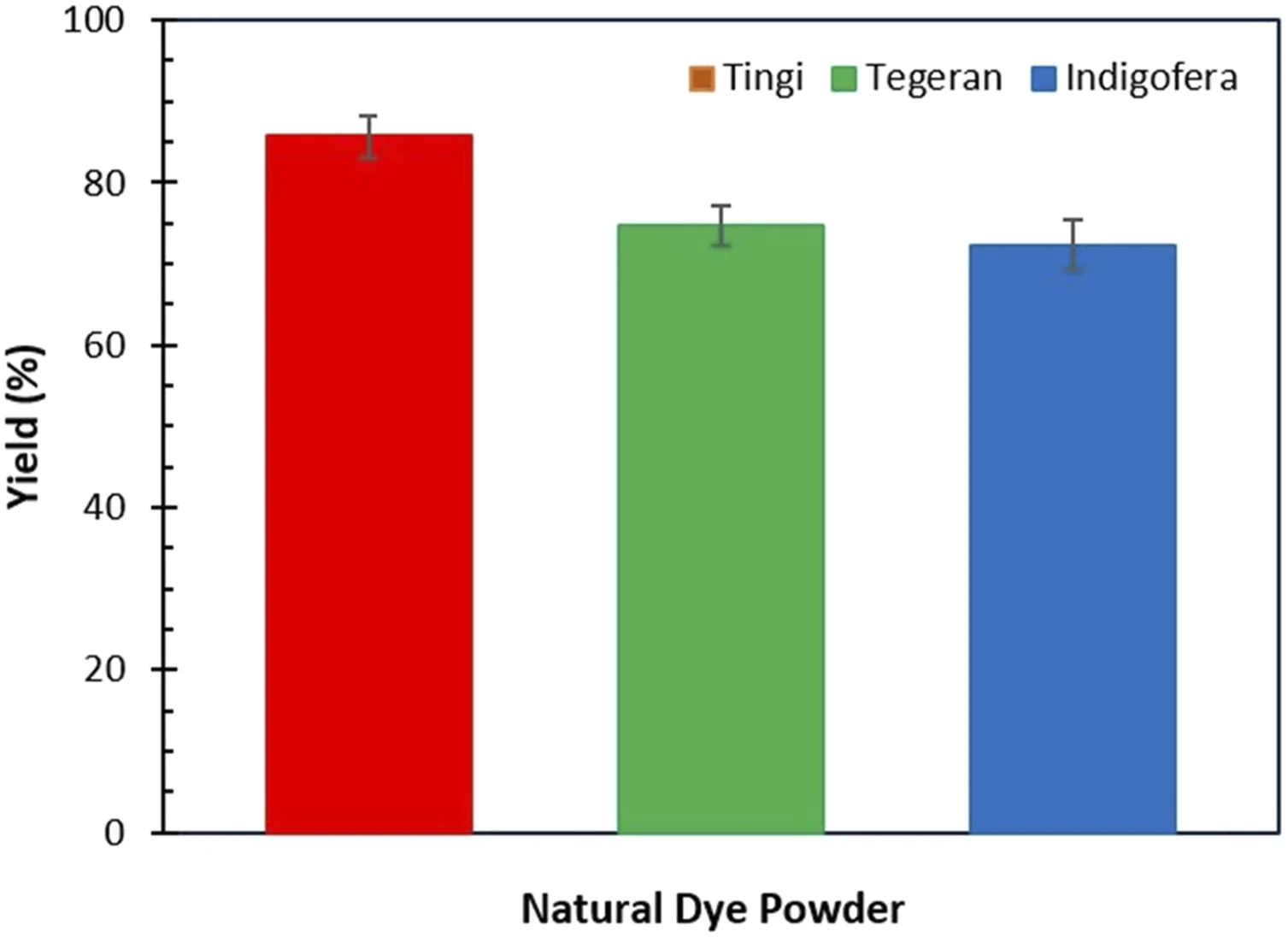
Yield of spray-dried natural dye powders from *Tingi*, *Tegeran*, and *Indigofera*.

In the spray drying process, the efficiency of powder production (yield) is a crucial indicator of the formulation and operational stability.^39^Fig. 1 shows the yield of natural dye powders from *Tingi*, *Tegeran*, and *Indigofera*. The findings revealed that the type of natural dye significantly affects the yield. The ANOVA showed an *F* value = 20.742 with *p* = 0.002 (*p* < 0.05), confirming the significant differences between the groups of dyes. The highest mean was obtained by *Tingi* (85.67%), followed by *Tegeran* (74.67%) and *Indigofera* (72.33%). The Tukey test revealed a significant difference in the yield of *Tingi* compared to those of *Tegeran* and *Indigofera*, while the yields of *Tegeran* and *Indigofera* were not significantly different. Technically, at the laboratory scale, yields greater than 50% are considered very efficient. It was also revealed that the combination of temperature and addition of 15% maltodextrin was able to overcome major physical obstacles such as surface adhesion. Lechanteur & Evrard^40^ reported that the efficiency of modern spray drying depends on the ability of the carrier agent to form a stable, homogeneous powder that is non-adhesive to the chamber wall.

The high yield of *Tingi* is scientifically driven by the dominant chemical characteristics of condensed tannin. The complex polyphenol structure of tannin has a relatively high glass transition temperature. The types of carriers and composition of raw materials are very important in determining the physical characteristics of the resulting powder. In this case, the interaction of tannin and maltodextrin accelerates the crust formation of the liquid drops, which avoids wall deposition.^41–43^ Moreover, maltodextrin serves to increase the total soluble solids, which is a key factor in stabilising the polyphenol compounds during the fast evaporation process.^39,40,44–46^

On the contrary, the relatively lower yields of *Tegeran* and *Indigofera* could be explained through particle dynamics in the cyclone. *Tegeran* contains a higher proportion of flavonoids, which are generally more hygroscopic than tannins. This may promote lump formation and deposition on the dryer surface, thereby decreasing overall yield.^47,48^ Meanwhile, the low yield of *Indigofera* (72.33%) is related to the characteristics of the indigotin pigment, which tends to form a suspension rather than a true solution. The existence of solid particles that are not completely dissolved resulted in an uneven particle size distribution. The fine particles fell short of the centrifugal force of the cyclone and were wasted with the exhaust air, thereby decreasing the yield.^49,50^

The utilisation of 15% maltodextrin supported the powder formation and improved handling characteristics under the conditions used in this study. However, its role in colour stability and its effects on storage and textile performance require further evaluation. It was confirmed that the addition of a drying agent such as maltodextrin is essential for bioactive-rich products to maintain their functional characteristics as well as to improve the ease of powder handling.^51^ Attaining a stable yield in the range of 72–85%, this formulation demonstrated initial potential for further development. However, evaluations of the process scale and textile application performance are needed.

### 3.2. Colour strength

**Fig. 2:**
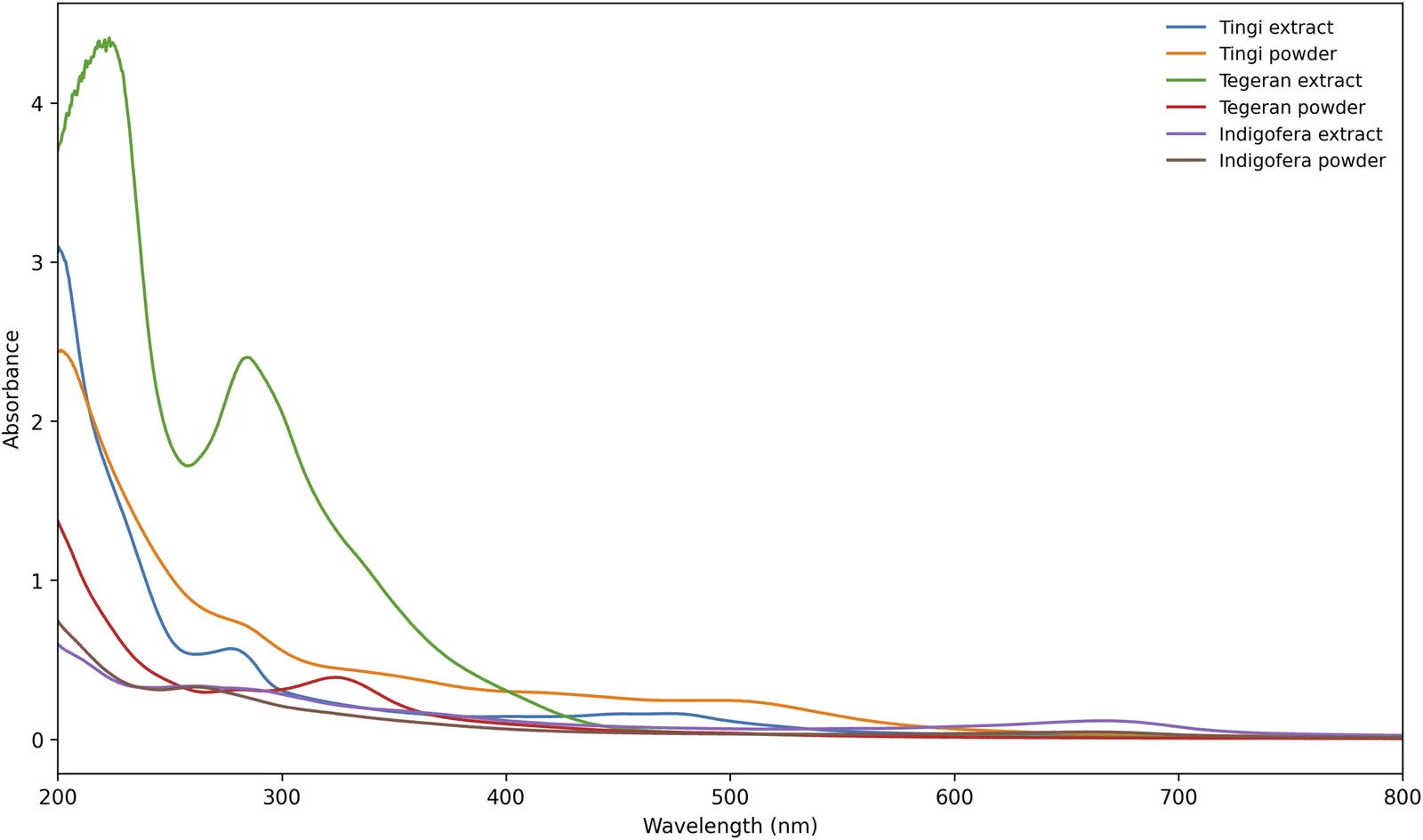
Average UV-vis absorption spectra before (untreated dye extracts) and after spray drying with the incorporation of 15% (w/w) maltodextrin as a carrier (dye powders) obtained from *Tingi*, *Tegeran*, and *Indigofera*.

The UV-vis absorption spectra for the three natural dyes are given in Fig. 2. The analysis of UV-vis spectrophotometry on the 15% maltodextrin-encapsulated natural dye powder provided a comprehensive description of the active compound retention after the spray drying process.^52^ In the standardisation framework of textile dyes, the main parameter evaluated is the ability of the encapsulation matrix to maintain the integrity of the chromophore structure, which is a part of the molecule responsible for light absorption, from thermal stress during the atomisation process. The statistical analysis confirmed a significant difference in the characteristic absorbance values among the natural dyes. The Kruskal–Wallis test showed *p*-value = 0.027 (*p* < 0.05), indicating that the type of natural dyes significantly affected the measured absorbance intensity. Descriptively, the mean rank value showed that after spray drying, *Tingi* dye had the highest absorbance, followed by *Tegeran* dye, while *Indigofera* dye had the lowest absorbance.

UV-vis spectra before and after spray drying were compared to evaluate the characteristic changes in chromophore absorption by the drying process and maltodextrin addition. In *Tingi* dye, the fresh extract provided a main band at 277 nm and a weak band in the visible area of 452–473.5 nm. After the spray drying and reconstitution processes, the absorption characteristics in the corresponding region were visible, although they appeared as broad bands without sharp peaks. This indicated that the light absorption component remained in the powder. However, the intensity was determined by the maltodextrin matrix, reconstitution concentration, and possibility of particle scattering. Therefore, the UV-vis results suggest a complex relationship regarding the stability of the tannin chromophore against thermal or oxidative changes during the drying process.^41,53,54^

The fresh extract of *Tegeran* revealed strong peaks at 223.0 nm and 284.5 nm. After spray drying, the observed peaks shifted to 281.5 nm and 323.5 nm, respectively, with lower intensities. The decrease in intensity indicated environmental changes due to the micro-pigment, dilution effects, or physical interaction changes within the solid matrix due to the spray drying process and the presence of maltodextrin. Therefore, the UV-vis data of *Tegeran* were more precisely interpreted as indicating partial retention of the spectral characteristics, highlighting the specific limits of chromophore protection under maltodextrin conditions.^55,56^

*Indigofera* showed the lowest absorbance. The UV band of *Indigofera* at 262.5 nm was relatively maintained after spray drying, with an absorbance retention of 98.50%. However, the visible band related to the indigotin pigment shifted from 666.0 nm to 658.5 nm and experienced an absorbance decrease from 0.116 to 0.046, with a retention of 39.66%. The decrease could be attributed to the lower solubility of indigotin in water, the effects of particle scattering after reconstitution,^57,58^ and the possibility of micro-environmental changes due to the presence of maltodextrin.

Overall, the UV-vis data indicated that the primary absorption characteristics of the natural dyes remained detectable after spray drying. However, the intensity changes and peak shifts revealed only partial chromophore retention. Hence, the UV-vis data demonstrated the changes and the retention of spectral characteristics, rather than providing definitive evidence that the chromophore structure was completely maintained.^59^

The formulation containing 15% maltodextrin provided potential in maintaining some absorption characteristics of the natural dyes, especially in the more easily dispersed dyes. However, pigment stability and dyeing performance need to be confirmed through storage and application tests. On the contrary, the results for *Indigofera* dye offer a technical insight that the standardisation of suspension-based pigments requires the optimisation of additional formulations, such as the utilisation of dispersing agents to increase the functionality and colour strength of the products.^60^

### 3.3. Molecular composition

**Fig. 3:**
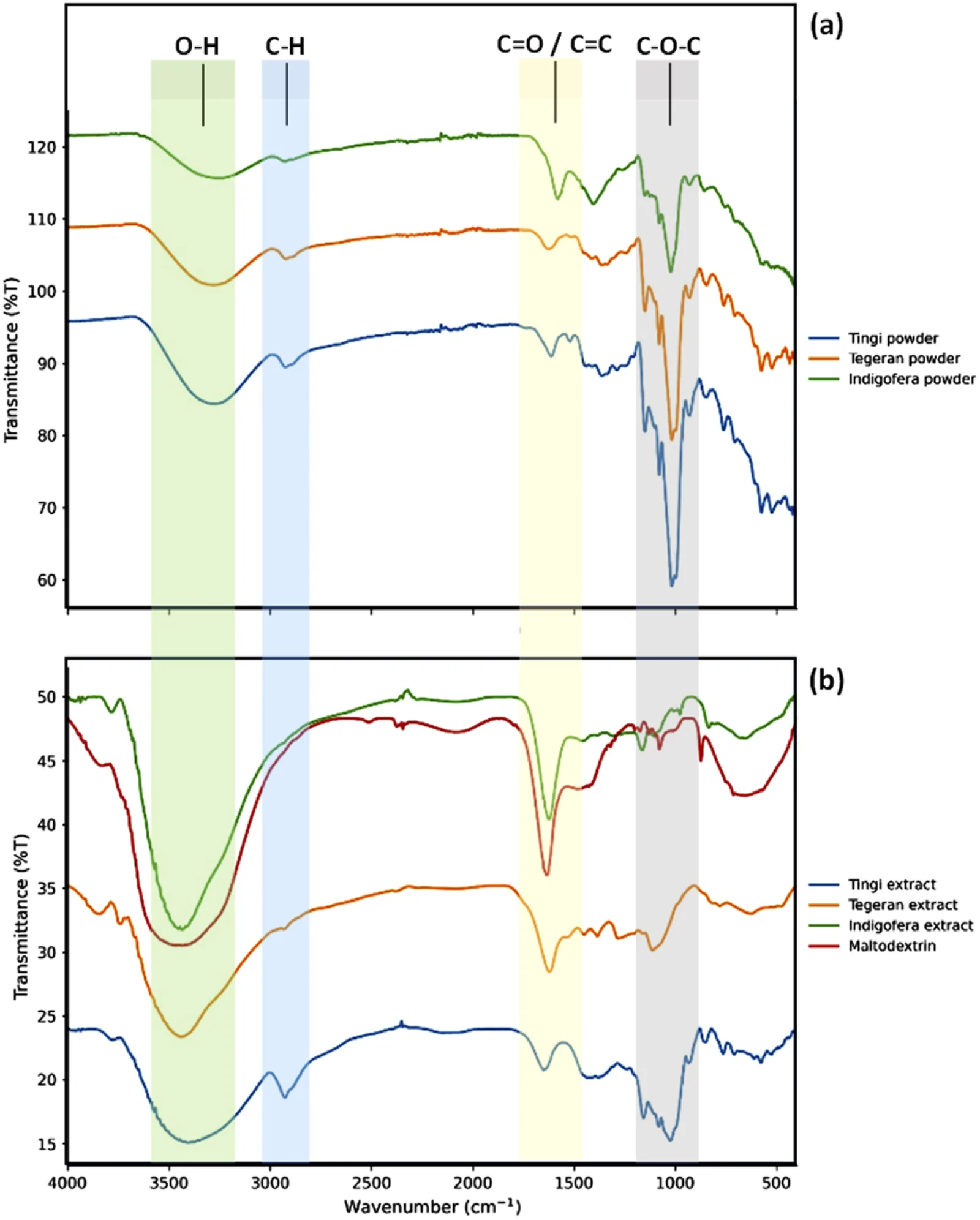
FTIR spectra of natural dye systems before and after spray drying: (a) spray-dried *Tingi*, *Tegeran*, and *Indigofera* powders formulated with 15% (w/w) maltodextrin; and (b) untreated dye extracts and pure maltodextrin reference spectra.

Fig. 3 presents the FTIR spectra of the natural dyes formulated using 15% maltodextrin after spray drying (a) and the pure dye extracts and pure maltodextrin before spray drying (b). FTIR was used to identify the main functional groups in the natural dye powders as well as to compare the spectral characteristics with those of maltodextrin and the dye extracts before the spray drying process. The comparison was utilised to determine the stability or changes in the major absorption band after the drying process, rather than to definitively prove the molecular interaction mechanism. The statistical analysis shows the significant differences of the three main absorption bands, *i.e.* 3400 cm^−1^ (O–H stretching), 1650 cm^−1^ (C=O or aromatic C=C), and 1080 cm^−1^ (C–O of polysaccharide) for the three natural dyes. The Kruskal–Wallis test confirmed that the three bands differed significantly between the types of dyes (*p* < 0.05). It indicates the variation in the molecular composition in the pigment system and the 15% maltodextrin matrix. The spray-dried powder spectrum indicated a strong contribution of the maltodextrin absorption band, especially in the C–O–C/C–O area. It revealed that maltodextrin was the dominant component and highly contributed to the powder matrix.

The wide absorption band of 3200–3600 cm^−1^ indicated the existence of hydroxyl groups (O–H) derived from maltodextrin or the phenolic compound of the *Tingi* and *Tegeran* extracts.^61^ Compared to the maltodextrin and dye extract spectrum before spray drying, the presence of the O–H band in the spray-dried powder revealed the presence of hydroxyl groups in the powder system. Changes in the form or the position of the O–H band could indicate the possibility of a physical association between the dye component and maltodextrin.^62,63^ The band in the range of 2850–2980 cm^−1^ was related to the vibration of aliphatic C–H, which could originate from the anhydroglucose unit of maltodextrin or the organic structure of the dye compound. The presence of this band in the powder spectrum indicated that the main organic component was still observable after the spray drying process. However, this data was not sufficient to conclude the absence of molecular fragmentation or oxidative degradation during drying.

The region of 1500–1650 cm^−1^ was used to observe the bands related to the aromatic structure of C=C or the carbonyl group of C=O. For *Tingi* and *Tegeran*, the bands in this region could be associated with the aromatic structures of tannin and flavonoid compounds, respectively, while for *Indigofera*, the bands in this region were related to the contribution of carbonyl groups in the indigotin structure. In the *Indigofera* sample, the specific absorption in the interval of 1620–1630 cm^−1^ represented the specific stretching of C=O (carbonyl) of the indigotin molecule. The unique identity of *Indigofera* was strengthened by the appearance of an absorption band in the range of 1300–1320 cm^−1^, confirmed as C–N stretching vibration or C–H bending of the *Indigofera* pyrrole ring. Overall, the presence of the specific bands in the powders revealed that the functional groups related to *Tingi*, *Tegeran*, and *Indigofera* were observable after spray drying.^64,65^

The dominant bands in the range of 1000–1150 cm^−1^ were associated with the C–O–C/C–O vibrations of the maltodextrin polysaccharide structure. The strong bands in this region indicated a high contribution of maltodextrin to the powder system. Comparison with the pure maltodextrin spectrum confirmed the presence of the maltodextrin matrix in the dye powder formulation. However, the band intensity of C–O–C is not an indicator of a uniform encapsulation wall, homogeneous carrier distribution, or complete protection against oxygen and humidity.^66,67^

Overall, the comparison of the FTIR spectra of the dye powder, maltodextrin, and the dye extract showed that the powder still contained the main bands associated with the dye components and the maltodextrin matrix. The absence of dominant new bands in the powder spectrum indicated no observed major spectral changes in the analysed FTIR regions. The O–H, C=O/C=C, and C–O–C bands revealed the possibility of physical association between the dye compounds and maltodextrin in the powder system. Therefore, FTIR was used as the basis for functional group identification and spectral change observation after spray drying rather than as direct evidence of bioactive protection or specific encapsulation mechanisms.

**Fig. 4:**
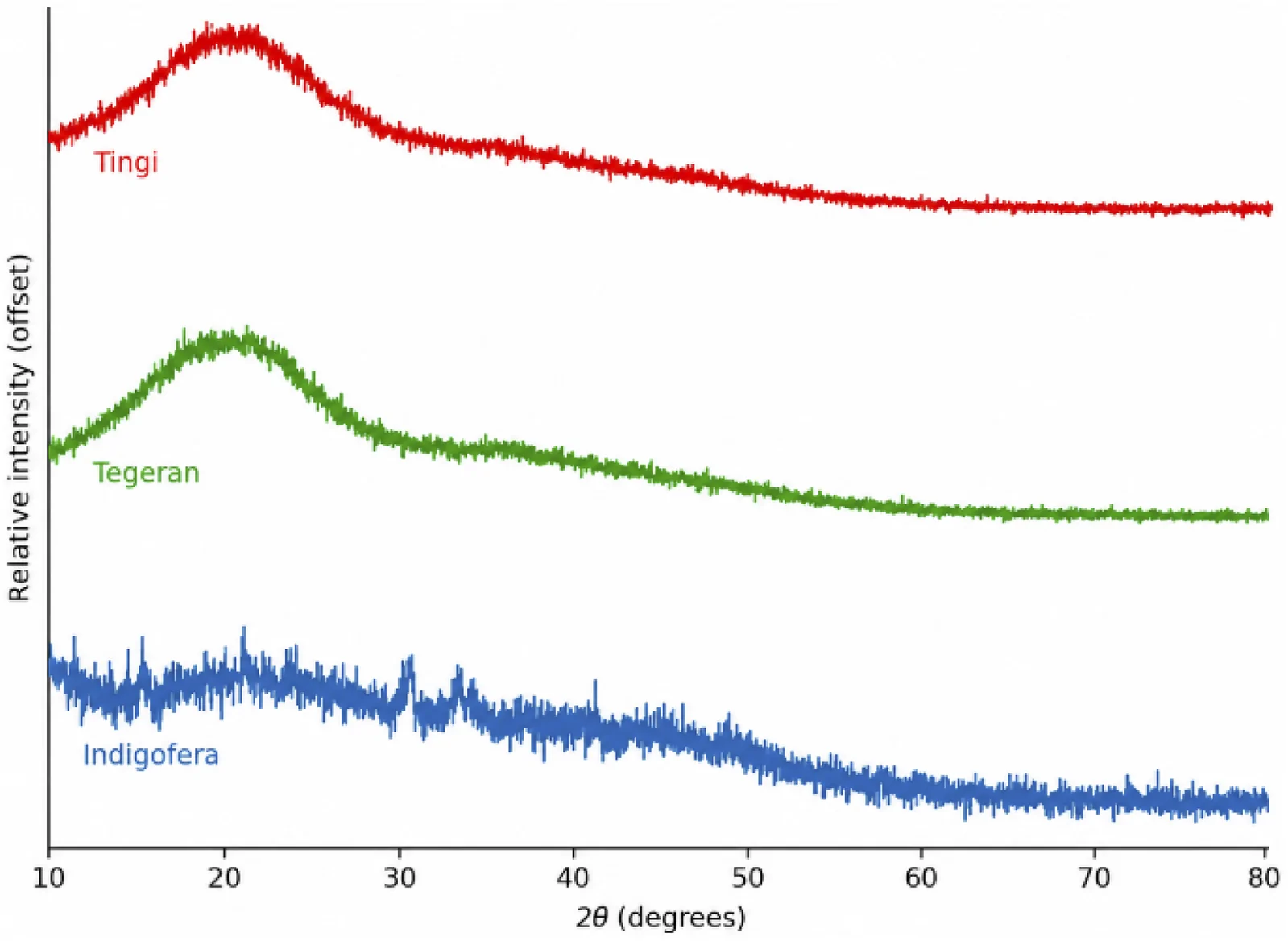
XRD patterns of spray-dried natural dye powders from *Tingi*, *Tegeran*, and *Indigofera* formulated with 15% (w/w) maltodextrin.

To support the FTIR interpretation, XRD analysis was performed to evaluate the characteristics of the spray–dried solid powder. The XRD results of *Tingi*, *Tegeran*, and *Indigofera* shown in Fig. 4 reveal the diffraction patterns dominated by broad halo patterns in the 2*θ* range of 18°–21°, without dominant sharp crystalline peaks. The patterns indicated that the spray-dried powder tends to have amorphous or semi-amorphous characteristics. The XRD results indicated the possibility of the formation of a maltodextrin-based irregular solid matrix, in which the pigment could be dispersed or physically associated. The combined FTIR and XRD data support the interpretation of amorphous/semi-amorphous matrix formation and possible physical pigment–carrier association.

### 3.4. Initial moisture characteristics and water activity

Moisture content and water activity (*a*_w_) are the initial parameters to describe the characteristics of residual water in spray-dried natural dye powders. Moisture content indicates the total water remaining in the powder, whereas water activity indicates the availability of unbound water that may influence moisture-related physical changes during storage, such as caking, stickiness, and flowability loss.^68^ These *a*_w_ values indicated that moisture-related physical risks should be considered, particularly under high-humidity storage conditions.^66^ The moisture content of the natural dye powders produced from *Tingi*, *Tegeran*, and *Indigofera* is presented in Table 1. The statistical analysis indicates that the type of natural dyes significantly affected the moisture content of the dye powder. ANOVA analysis resulted in *F* = 298.580 with *p* < 0.001.

**Table 1:** Moisture content and water activity of spray-dried natural dye powders

Sample	Moisture content (%)	Water activity
*Tingi*	11.27 ± 0.07	0.6033 ± 0.0007
*Tegeran*	10.31 ± 0.07	0.6059 ± 0.0015
*Indigofera*	9.87 ± 0.08	0.6436 ± 0.0030

The highest moisture content was observed in *Tingi* and possibly related to the characteristics of tannins and polyphenol compounds with hydrophilic groups that retain more residual water in the powder matrix. Therefore, the high moisture content of *Tingi* (11.27% ± 0.07%) needs to be taken into consideration when designing appropriate storage conditions. The value indicates potential risks of physical changes such as caking or decreased flowability.

*Tegeran* and *Indigofera* showed lower moisture contents than *Tingi*, *i.e.* about 10.31% ± 0.07% and 9.87% ± 0.08%, respectively. However, the water activity demonstrated a different trend. *Indigofera*, with the lowest moisture content, resulted in the highest water activity (0.6436 ± 0.0030. This phenomenon could be attributed to the fact that despite the low moisture content, some residual water might be available in a less strongly bound form in the powder matrix. It is possibly related to the characteristics of indigotin, which is less soluble in water and tends to remain as a suspended pigment. Therefore, the interactions of the pigment, maltodextrin, and water may differ from those in the polyphenol-rich dye system. The higher water activity of *Indigofera* indicates the need to consider the storage conditions, especially in high-humidity areas, to avoid the risks of physical changes.

As indicated in Table 1, the water activities of *Tingi*, *Tegeran*, and *Indigofera* were slightly higher than the threshold of 0.60. The presence of free water indicates the importance of humidity control and the use of airtight packaging. Accelerated or real-time storage studies are important to examine the long-term storage stability through the parameters of pigment degradation, colour intensity retention, caking, stickiness, and decreased flowability.

### 3.5. Particle size distribution

Particle size distribution analysis is needed because the particle size and distribution are important for understanding the physical characteristics of natural dye powders. The most significant parameters are the size heterogeneity, agglomeration tendency, and potential dispersion behaviour of the reconstituted powder. Particle size distribution could be affected by carrier characteristics and dye extract used in the formulation.

**Fig. 5:**
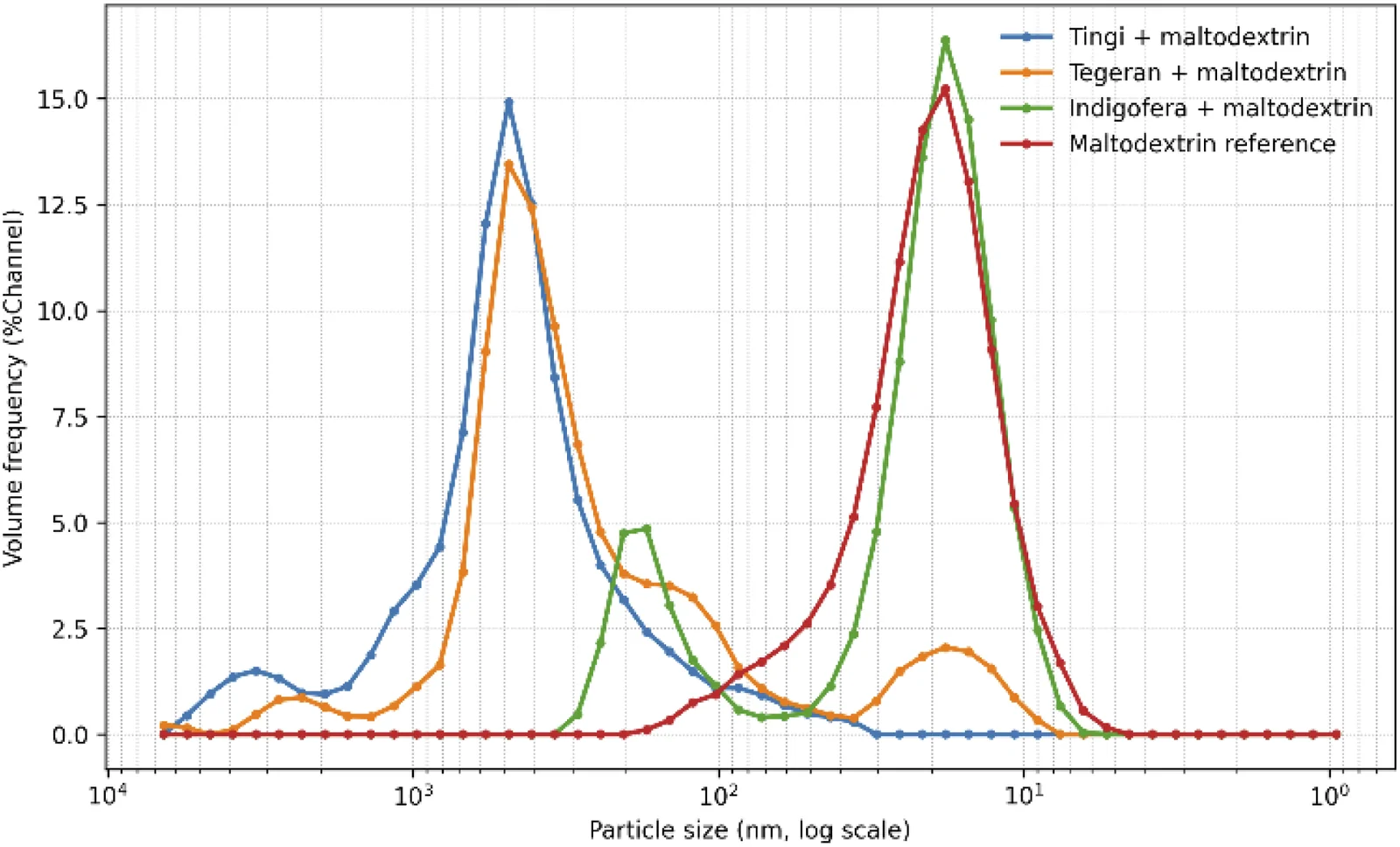
Particle size distribution (PSD) of spray-dried natural dye powders formulated with 15% (w/w) maltodextrin and maltodextrin reference. The distributions are presented as volume frequency (%Channel) *versus* particle size on a logarithmic scale to compare the particle size characteristics of *Tingi*, *Tegeran*, *Indigofera*, and the maltodextrin reference.

Particle size distribution profiles of *Tingi*, *Tegeran*, *Indigofera*, and the maltodextrin reference are presented in Fig. 5. To support the comparison, the main particle size parameters of the maltodextrin reference are summarized in Table 2. Particle size distribution is an initial physical parameter used to represent the particle formation and size heterogeneity of spray-dried natural dye powders. The statistical analysis indicated that the particle size of each type of dye was significantly different. ANOVA analysis resulted in *F* = 53.802 with *p* < 0.001, indicating a significant difference in particle size between the groups. Based on the experimental data, the natural dye powders of *Tingi*, *Tegeran* and *Indigofera* exhibited a comprehensive distribution profile in the interval of 6.39 to 6540 nm. The size interval revealed a wide size distribution, ranging from nanometres to micrometres.

**Table 2:** Particle size characteristics of the maltodextrin reference

Sample	Main peak/D50 (nm)	D90 (nm)	PDI
Maltodextrin 1	17.73	45.10	0.1984
Maltodextrin 2	18.69	39.00	0.2131
Maltodextrin 3	18.97	48.10	0.1739
Mean ± SD	18.46 ± 0.65	44.07 ± 4.61	0.1951 ± 0.0197

The particle distributions of *Tingi* and *Tegeran* indicated relatively similar patterns with a dominant population in the submicron range. *Tingi* had a dominant particle size in the range of 409–578 nm, while *Tegeran* showed a dominant particle size in the range of 344–486 nm. Compared to maltodextrin as a reference, both dye powders experienced a shift to larger particle sizes. This condition indicated that the presence of the tannin-rich *Tingi* extract and flavonoid-containing *Tegeran* extract was possibly related to the formation of larger particles or agglomerates during the spray drying process.

A different pattern appeared in the *Indigofera* sample with a dominant particle size of about 18.06 nm. The Tukey test revealed a significantly different particle size of *Indigofera* than those of *Tingi* and *Tegeran*. The dominant size was close to the main peak of the maltodextrin reference; thus, the fine particle fraction of *Indigofera* powder was probably affected by the maltodextrin matrix. Due to the low solubility of indigotin in the *Indigofera* extract, which tends to be in a suspension form, the particle size distribution needs to be interpreted as a mixture of the maltodextrin matrix and suspended pigment particles rather than as pure pigment particles. The fine particle fraction may also contribute to the low yield because these particles are more easily carried away by the outgoing air flow during the cyclone separation process.

Theoretically, the existence of a smaller particle fraction could support the dispersion process during reconstitution. However, a smaller particle size cannot always be interpreted as a functional benefit. Smaller particles have a relatively larger surface area and thus can enhance the contact with moisture and oxygen. Therefore, a fine particle fraction also has the potential to increase risks of water absorption, caking, stickiness, or pigment oxidation during storage. In this study, the particle size distribution is interpreted as an initial physical characteristic of the powder, rather than as direct evidence of stability or functional performance improvement.

The results of particle size distribution need to be interpreted along with those of moisture content and water activity. At a water activity of about 0.60, the powder still has residual water content, and thus, the existence of fine particles must be practically considered for storage. This is especially relevant for *Indigofera*, which had the highest dominant fine particle fraction and water activity value. This finding indicates that the particle size, moisture content, and water activity must be jointly considered when evaluating the physical handling characteristics of spray-dried natural dye powders.

### 3.6. Particle morphology

**Fig. 6:**
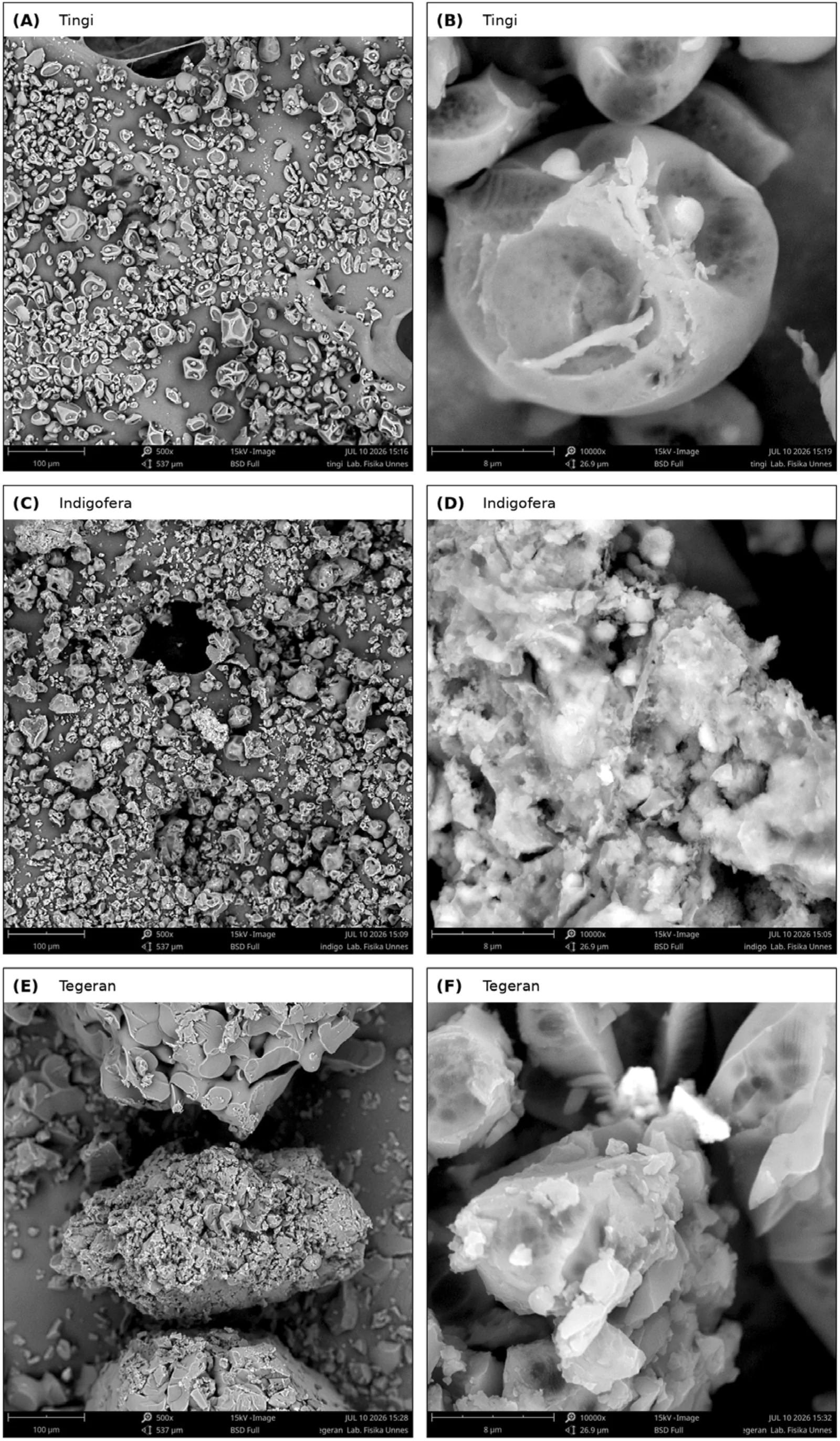
SEM micrographs of spray-dried natural dye powders formulated with 15% maltodextrin: (A and B) *Tingi*, (C and D) *Indigofera*, and (E and F) *Tegeran* at magnifications of 500× and 100 00×, respectively.

SEM characterisation was performed to evaluate the morphology of the spray-dried natural dye powders with the addition of 15% maltodextrin. This analysis provides visual information about the particle shapes, surface conditions, and agglomeration tendencies that complement the particle size distribution results. SEM micrographs of *Tingi*, *Indigofera*, and *Tegeran* powders formulated with 15% maltodextrin are displayed in Fig. 6.

The SEM results of *Tingi* powder showed round to irregularly shaped particles. The particles appeared hollow, concave, or collapsed. The morphology indicated the formation of a particle structure during the spray drying process, which was likely affected by the formation of the outer layer of maltodextrin and the rapid evaporation of water. The presence of hollow particles and surface cracking indicated particle shrinkage during the drying process.

Meanwhile, the *Indigofera* powder revealed a more irregular morphology with a tendency for agglomeration. The particle surface was rough, with smaller particles stuck to larger particles. This condition could be related to the low water solubility of indigotin, which exists mostly in a suspension form. Therefore, the *Indigofera* morphology supports the interpretation that after spray drying, the suspension-based pigment system tends to form a less homogeneous particle distribution.

*Tegeran* powder revealed a higher level of agglomeration with a rough surface and flat particle fragments. Smaller particles stacked on the agglomerated surface indicated interparticle cohesion after the drying process. The morphology determines the dispersion capacity and thus needs to be considered in the development of natural dye powder formulations.

Overall, the SEM results showed that each dye exhibited different morphological characteristics despite the same maltodextrin concentration and spray drying conditions. This indicated that the intrinsic properties of the pigment, such as solubility, chemical composition, and initial dispersion form, also influence particle formation. Therefore, SEM data support the PSD results and provides visual evidence that the morphology of particles must also be considered in the standardisation of natural dye powders.

## 4 Conclusion

Based on the results, it could be concluded that the spray drying process with the addition of 15% maltodextrin produced natural dye powders with measurable initial physicochemical characteristics. The process produced yields ranging from 72.33% to 85.67%, with *Tingi* showing the highest yield. The powders had residual moisture contents of 9.87–11.27% and water activity values of 0.6033–0.6436. These values should be interpreted as initial moisture-related characteristics and potential indicators of moisture-related risks. The UV comparison of the fresh extract and the reconstituted powder solution demonstrated partial retention of the chromophore absorbance characteristics despite changes in absorbance intensity and peak shifts in some samples. The FTIR results also indicated the contribution of maltodextrin to the formation of the solid matrix and the possibility of a physical association between the pigment and carrier. However, the combined FTIR and XRD data should not be interpreted as definitive evidence of specific hydrogen bonding. Physically, the resulting products had wide size ranges of 6.39–6540 nm. The PSD and SEM results indicated differences in particle size distribution and morphology (including fine particles, irregular shapes, collapsed particles, rough surfaces, and agglomeration tendency) among the three dye powders. These characteristics are relevant for understanding the powder handling and dispersion behaviour, but they do not directly demonstrate the textile dyeing performance. Overall, this study provides initial physicochemical information on the spray-dried natural dye powders formulated with 15% maltodextrin. Further accelerated or real-time storage studies and textile dyeing performance tests, including colour strength and colour fastness evaluation, are still required before industrial textile application can be claimed.

## Author contributions

AK: conceptualization, methodology, investigation, formal analysis, writing – original draft, supervision, funding acquisition. SA: methodology, investigation, formal analysis, writing – review & editing. OP: formal analysis, validation, writing – review & editing. RM: investigation, data curation. OBS: conceptualization, methodology, writing – review & editing, supervision.

## Conflicts of interest

There are no conflicts of interest to declare.

## Data Availability

The data supporting the findings of this study are available within the article, including all data presented in the figures and tables.
