# Correction: Standardisation of natural dye powders *via* spray drying: initial physicochemical characterisation

**DOI:** 10.1039/d6ra90156e

**Published:** 2026-10-22

**Authors:** Adhi Kusumastuti, Samsudin Anis, Octavianti Paramita, Retno Marlangen, Ooi Boon Seng

**Affiliations:** a Faculty of Engineering Universitas Negeri Semarang, Kampus UNNES Sekaran Gunungpati 50229 Semarang Indonesia adhi_kusumastuti@mail.unnes.ac.id; b School of Chemical Engineering, Engineering Campus Universiti Sains Malaysia 14300 Nibong Tebal Pulau Pinang Malaysia

## Abstract

Correction for ‘Standardisation of natural dye powders *via* spray drying: initial physicochemical characterisation’ by Adhi Kusumastuti *et al.*, *RSC Adv.*, 2026, **16**, 51089–51098, https://doi.org/10.1039/d6ra04201e

The authors apologise for supplying incorrect funding information in the ‘Acknowledgments’ section. The correct funding information is as follows:

The Royal Society of Chemistry apologises for these errors and any consequent inconvenience to authors and readers.

